# A Golden Age for Motor Skill Learning? Learning of an Unfamiliar Motor Task in 10-Year-Olds, Young Adults, and Adults, When Starting From Similar Baselines

**DOI:** 10.3389/fpsyg.2020.00538

**Published:** 2020-03-25

**Authors:** Marius Solum, Håvard Lorås, Arve Vorland Pedersen

**Affiliations:** ^1^Department of Neuromedicine and Movement Science, Faculty of Medicine and Health Sciences, Norwegian University of Science and Technology, Trondheim, Norway; ^2^Department of Teacher Education, Faculty of Social and Educational Sciences, Norwegian University of Science and Technology, Trondheim, Norway

**Keywords:** skill development, skill acquisition, sensitive period, critical period, coordination

## Abstract

It is often argued within sports circles that the age span of around 6–12 years is a *golden age* for motor skill learning, and this period is often described as sensitive, or even critical, for learning such skills. Consequently, skill development programmes target this age span for teaching technical and coordinative skills. In the scientific literature, however, the term *golden age* is scarcely seen, and few studies have even attempted to test this hypothesis. When comparing motor learning between children and adults, studies have typically found little difference or differences favoring adults. Studies that have reported precocious learning within the golden age seem not to have controlled all relevant variables. Typically, the different age groups have not started from similar baselines and have tested tasks that have not been scaled according to physical differences between individuals belonging to the various groups. The present study tested 10-year-olds, 18-year-olds, and 40-year-olds on dart throwing with their non-dominant hands. They each completed 200 throws over 2 days, with 1 day in between. All participants performed at similar levels at the pre-test, and the task was scaled according to each participant’s individual size. No difference was found between the groups after practice in terms of change in absolute error, or with respect to the slopes of their learning curves. The 10-year-olds’ learning curves were more variable compared with the other groups. Thus, the present study found no evidence that the 10-year-olds belonged to a *golden age* for motor learning, and we would argue that previous findings of differences might well be artefacts due to lack of control of relevant variables.

## Introduction

Terms like a *golden age of motor skill development/learning* (e.g., USA Hockey, American Development Model, see Mancini, 2015; Squillante, 2018; Parisi, 2019; Super Futsal School Program, 2019) or similar concepts are frequently used within sport settings in the context of development and learning of motor skills. It is often claimed that the basis for later success is established within this supposed golden age, and sometimes also that it will be difficult to learn certain skills once the golden age has passed. Some would argue that what has not been developed within the golden age can never be made up for later in life (e.g., Hefti, 2007). Talent development programmes and the like base their developmental models on the notion that children within the golden age are “in prime position to develop motor coordination and acquire sport-specific skills” (USA Hockey, American Development Model; Mancini, 2015), or that the golden age constitutes a “window of optimal trainability” or “… sensitivity for skill training” (Skate Canada, 2010, both on p. 9).

There seems to be less agreement as to what exact age is the golden one. USA Hockey (2015) state, for instance, it is 9–10 years. Skate Canada (2010) says 8–12, while the Super Futsal School Program (2019) has decided the golden age lies between 6 and 12 years. The majority of estimates seem to lie within the range of 6–12 years, with the majority of authors agreeing that the onset of puberty closes the period. Some writers define different golden ages for boys and girls accordingly (e.g., Squillante, 2018; Parisi, 2019).

Despite being widely used within the more practical domains, such terms as a golden age are rarely found in the scientific literature, with a few notable examples (e.g., Hirtz and Starosta, 2002; Di Cagno et al., 2014; Musalek, 2015; Read et al., 2015).

The exact origin of the concept, or the precise mechanism underlying it, remains unclear, and few of the sources present actual data or cite others that have presented data. Regardless, the term has been reiterated to the extent that it has become something of a neuromyth (see Bailey et al., 2018, for a discussion of such).

The scientific term that comes closest to a golden age by the strictest definition of the term (i.e., no learning will occur outside the period) would be a *critical period*. This term was coined by Lorenz (1937) based on studies of the association of new-born animals with their parents. Lorenz held that such a period is critical in that while within it one will learn skills that are crucial for subsequent skill learning. Within a critical period, one will learn skills faster than later in life, and one will acquire skills that are irreversible (Bateson, 1983; Bornstein, 1989; Knudsen, 2004; Thomas and Johnson, 2008). A concept that moderates the *critical period* and may thus be more similar to a *motor golden age* as described above, is the *sensitive period*. Learning occurs at a faster rate within a sensitive period compared with other periods in life (Bateson, 1983; Knudsen, 2004). This increased ability to learn, Knudsen argued, is due to the fact that the brain’s plasticity is greater in the sensitive period; thus, it changes faster based on experience. Consequently, the brain is more receptive to experiences during this period. The brain will develop new neural circuits in the sensitive period that form the bases for further learning later in life. In addition, individuals will develop stronger synapses between nerve cells as well as getting rid of those that are less adaptive (Knudsen, 2004).

An oft-cited paper by Hirtz and Starosta (2002) argued the existence of sensitive (and even critical) periods of motor co-ordination development and presented, together with some anecdotal evidence, data from longitudinal learning studies as support for their argument. The data, however, would seem rather limited as support for a sensitive period, as they simply showed learning in individuals from the ages of about eight until 15 years, presenting learning curves that were steeper in the beginning of the period. The learning curves, thus, were rather similar to those found in any learning study, at any period in life (e.g., Karni et al., 1998; Newell et al., 2001; Wulf et al., 2010). Hirtz and Starosta (2002), nevertheless, concluded that coordination could be trained particularly well in the period before puberty and that this period should be especially targeted for such training.

A strand of studies have compared the learning of motor skills in adults and children by means of a motor-sequence learning paradigm, in which participants are presented images on a computer screen and asked to press a key corresponding to the visual cue that appeared on the screen while their reaction times and the accuracy of their responses were measured (Meulemans et al., 1998; Thomas and Nelson, 2001; Thomas et al., 2004; Savion-Lemieux et al., 2009). Schmitz and Assaiante (2002) used a slightly different task – load-lifting – but also measured reaction time. The general trend of results was that the adults improved less compared with children, while Du et al. (2017) found that both children and adults learned motor sequences quickly, within a single session, albeit displaying somewhat different learning processes. However, in Du et al. (2017) no differences were found favoring either age group.

In the above-mentioned studies, the different groups of participants had practiced equally. However, adults generally demonstrated a floor effect, as they had little room for improvement, performing close to the floor already at the pre-test. It is important to emphasize that although the tasks showed a difference in the learning curves for adults and children, there were only a few significant differences and some tendencies of difference between learning curves in the studies. Furthermore, their tasks were not sport-specific or even particularly sport-like, with performance on a serial reaction task being determined mainly by a limited motor response in the form of pushing a button after sometimes serious cognitive demand (Robertson, 2007). Thus, they were not necessarily very relevant for the present purpose of studying movement skills associated with the putative *golden age*, most often gross motor skills involving coordination of a number of muscles or even whole-body movement, as is typically seen in sports. As Robertson also stated, in his oft-cited review of the subject, “Although the SRTT is often viewed as a motor learning task, it is not clear that learning is taking place solely within the motor domain” (p. 1074).

Janacsek et al. (2012), also studying motor-sequence learning, compared individuals across the whole lifespan, and found the strongest learning effect in the 4-to-12-year-old age group. Also, in this study, the differences in baseline skill posed a challenge for the interpretation of the results, and the authors discussed whether the children’s large improvement could in fact be explained by their slower reaction times at baseline.

Lukács and Kemény (2015) challenged the view that childhood would necessarily be the prime period for learning skills. They used a similar paradigm as Janacsek et al. (2012), and tested individuals across the whole lifespan on, among other learning tasks, a motor-sequence learning task. However, they corrected for baseline differences by comparing normalized data and found that learning was in fact more effective in adolescence than in childhood. In addition, adult groups learned better than children, and a decline was seen only in older adults.

A few studies stand out as somewhat different from those already mentioned in that they included more sport-like or whole-body motor learning in contrast to the motor-sequence learning paradigm with rather simple motor responses to cognitive stimuli. Lee et al. (2018) showed that children – both 9-year-olds and 12-year-olds – who learned to control a cursor on a computer screen by means of upper-body movements performed poorer compared with adults. The results further indicated that the reason for the poorer performance was that children did not explore their movement repertoires to the same extent as did the adults. Hence, the children demonstrated a more limited movement repertoire, with less variability.

Emanuel et al. (2008) tested children and adults who learned to play darts and found no general difference in learning between the groups. If anything, the differences favored the adults. In Voelcker-Rehage and Willimczik (2006) individuals belonging to different age groups between 5 and 80+ years learned a juggling task. The results showed that the youngest children (an age group corresponding to the putative golden age) had, in fact the poorest performance of all age groups and were outperformed by even the oldest adults.

Finally, Schärli et al. (2013) tested adults and 8-year-olds on a dynamic balance task that was unfamiliar to the participants, namely balancing on a slackline. In Schärli et al., adults learned faster than the children. Furthermore, the children had greater ranges of motion than the adults did when they balanced on the line, which is seen as a less efficient strategy, making it harder to recover a loss of balance. Thus, Schärli et al.’s results did not support the assumption of a golden age that includes 8-year-olds. However, not knowing that the groups started from a similar baseline, the results cannot support a difference in learning favoring either group.

As is evident, a common problem with previous studies has been that the groups have started out from different baselines, and so they have appeared to have different learning profiles. Furthermore, adults have typically exhibited ceiling/floor effects (depending on the direction of the relevant variable) because they were already superior in the skill. Thus, it has appeared that children learn quicker than adults simply because their baseline is different. In general, based on the above review of the rather scarce literature, there seems to be little support for a golden age in prepubescent children, most often defined to be between 7 and 12 years (e.g., Hirtz and Starosta, 2002).

The present study was performed in an attempt to shed more light on the question by at least eliminating some of the previous methodological shortcomings. More specifically, this was done by securing that all participants would be performing at comparable skill levels at the onset of the study, by choosing a task demanding limited cognitive abilities and by scaling the task so that it would not particularly favor any of the included age groups. Perhaps most important, the task was performed with the non-dominant hand, thus further interrupting the usual performance. The present research question was this: Will children within the age span of a putative *motor golden age* show precocious motor learning relative to individuals outside the *golden age* when practicing relatively the same novel task equally well and when starting from similar baseline skill levels?

## Materials and Methods

### Participants

The present study included three groups of healthy participants: one that represented the *golden age* – decided to be 10-year-olds as they would seem to be included in whatever age span that has been argued to constitute such a *golden age*. They were compared with participants outside the *golden age*, namely 18-year-olds. Furthermore, a group of adults were tested, who were at the age of 34–45 years old, that is, nowhere near the *golden age*. In total, 27 individuals participated, including nine 10-year-olds [mean (SD) age: 10.35 (0.25) years], eight 18-year-olds [mean (SD) age: 18.75 years (0.36) years] and ten adults [mean (SD) age: 39.76 (3.30) years]. All participants signed an informed consent form – with parents signing for the 10-year-olds – after receiving written information about the study. The study protocol was approved by the Norwegian Centre for Research Data.

### Matching Groups on Baseline Skill Level

Potential participants answered a questionnaire asking about any prior experience with dart throwing or with activities that could resemble dart throwing. Furthermore, their handedness was determined by means of the Edinburgh Handedness Inventory (Oldfield, 1971). In order to ensure comparable skill levels at baseline across groups, the average result of the first 15 throws for each participant was calculated. Potential participants were excluded, when they at baseline performed differently than the combined average for the total pool of participants (all three groups), either better or poorer. In this selection process, eight individuals were excluded. This secured three groups that were matched on baseline skill, something that had been lacking in previous, similar studies.

### The Task to Be Learned

Dart throwing was used as the task in the present study. As it was easy to accumulate a sufficient number of repetitions within a relatively short time, it could ensure a similar baseline regardless of age, and has, as mentioned in the introduction, proved to be feasible for both adults and children in previous learning studies (e.g., Emanuel et al., 2008). It’s feasibility, at least in part, might be due to the fact that it demands rather limited cognitive abilities. Furthermore, the performance can be easily measured on a ratio scale, and it can be scaled according to physical differences across groups (most notably, differences in size). The task is difficult enough that it is unlikely that any participant will demonstrate a floor level, thus it was possible to recruit participants of similar skill level across different age groups.

The task would be similar to many of those sport-specific techniques that are practiced within the putative “golden age.” In fact, darts *is* a sport and shares important characteristics with many other sports, most notably the coordination of a number of muscles and muscle groups in order to produce movements of the arm and hand that balances accuracy in relation to force, while simultaneously stabilizing and balancing the body. Furthermore, such factors as concentration and visual acuity may affect the result (Nasu et al., 2014; Tran et al., 2019).

Even though few children play darts competitively, family dart sets are available in any toy store, as well as in supermarkets and roadside petrol stations, to the extent that one would be hard-pressed to find any individual, at least in developed countries, who had no concept of the skill of dart throwing. True, even without any specific instruction about throwing technique, all participants intuitively used the same general (proper) overarm throwing technique that is typical of darts (Nasu et al., 2014; Tran et al., 2019).

In order to maximise the similarity of baseline performances and to enhance the possibility of increased rate of improvement, thus making it easier to spot differences across groups, participants threw with their non-dominant hands, as such throws are usually much less accurate than dominant-hand throws, and also more variable (Hore et al., 1996). The non-dominant hand was, for all participants, their left hand as determined by the Edinburgh Handedness Inventory. Non-dominant hand throwing was also deemed feasible for studying how the learning process might be different, since the scope was not to study *whether* the participants would demonstrate motor learning, but rather to study *how* they would learn across the three age groups. This would have been much more difficult to obtain had participants used their dominant hands, since most adults have some experience in playing darts with their dominant hand and may have more experience with other similar right-hand tasks. Feasibility of the task was further indicated by the fact that the three groups, in fact, performed similarly at baseline.

The distance to the dartboard was scaled according to participants’ height, and the centre of the dartboard was positioned level with each participant’s eyesight. The formula used to calculate the distance to the dartboard was the participant’s height of eyesight × 1.5, effectively the same as the standard dart-throwing distance for an individual of around 1.6 m in height.

To establish the learning curves, the distance from the impact point to the centre of the dartboard was measured, in millimetres, and thus resembles the absolute error. All throws for each participant were logged, thus providing enough data for analyzing the slopes of the learning curves and their possible change. A total of 200 throws were completed for each subject, spread equally across 2 days.

### Equipment

A standard-sized dartboard was used, with the centre point (bull’s-eye) as the aiming point. The board had ten alternating yellow and black concentric circles numbered from one to nine, and with the bull’s-eye indicating ten points. The dartboard hung on a Styrofoam wall with the dimensions 180 cm × 240 cm, into which the darts stuck easily. This ensured that it was easy to measure the outcome in those cases when the darts did not actually hit the dartboard. A pulley with a rope was attached to the dartboard, enabling individual adjustment of the height of the dartboard for each participant.

**FIGURE 1:**
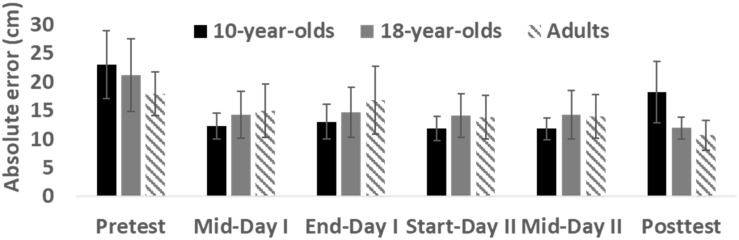
Absolute error at different timepoints across study groups. Bars depict mean and error bars SD.

Dartboards are designed so that a dart will remain embedded regardless of the force of the throw (Tempest, 1937). Thus, the distance from the centre point to the point of impact (i.e., the absolute error) could easily be measured after each throw. The only time the dart was not stuck into either the wall or the dartboard was when it hit the metal rim around the dartboard. In such cases, the distance from the centre point to the metal rim, measured in advance, was given as the result. The weight of the darts could not be aligned across groups without changing their performance significantly. However, it is reasonable to assume that the relative difference was small enough so as not to affect the results, an assumption that was supported by the fact that groups performed similarly on the pre-test, with no significant differences in skill level amongst the groups at this stage (Figure 1), and the pre-test results indicated further that no floor effect would likely be demonstrated by any individual, or at group level.

### Cognitive Considerations

Participants received a minimum of instruction. No verbal feedback was given. Thus, participants learnt by trial and error, discovering for themselves the best throwing strategy. The darts were presented to all participants in a similar manner before each throw so as not to affect their choice of strategy. Participants had some visual feedback available as they could monitor the success of each throw visually. However, their visual feedback was limited by removing the darts after each throw. Two participants were present in the room at the same time but were not able to observe each other’s throwing or to monitor each other’s results. The only instruction given was that they should aim at the centre of the dart board (the bull’s-eye). They were provided with no instructions whatsoever about which technique or strategy to choose. Still, all the included participants spontaneously chose the same (proper) overarm throwing motion as is common in dart throwing.

### Statistical Analysis

Kolmogorov–Smirnov tests, histograms, and Q–Q plots were examined and indicated that normality assumptions of the variables were not met. Thus, non-parametric tests were applied. The average result for each participant’s first 15 throws was used as a pre-test, while the average of the last 15 throws constituted the post-test. Between-group differences in pre-test, post-test and improvement (change) in skill from pre- to post-test was examined with Kruskal–Wallis one-way ANOVAs, with eta squared (η^2^) as a measure of effect size. In case of significant ANOVAs, further post-hoc analysis was conducted with Mann–Whitney *U* tests. Within-group analysis of change in dart-throwing accuracy was examined by paired Wilcoxon signed-rank tests.

Furthermore, individual learning curves were subjected to several statistical analyses, testing changes in performance across each participant’s 200 throws. First, possible between-group differences in variability of learning curves (standard deviation) were examined with Kruskal–Wallis ANOVAs and Mann–Whitney *U* tests. The average learning curves from each study group were uploaded to MATLAB R2019a v. 9.6.0 (MathWorks, Natick, MA, United States) and compared by cross-correlation analysis (*r*) after first applying a moving average filter with a sliding window of 10 to each learning curve. Furthermore, the slope of each individual learning curve was extracted by linear regression analysis and compared between groups by Kruskal–Wallis ANOVA. Also, the average absolute error at midday I (trials 50–60), end-of-day I (trials 90–100), beginning-of-day II (trials 100–110), and midday II (trials 150–160) was subjected to between-group analysis by means of a Kruskal–Wallis ANOVA. Statistical analysis was conducted using PASW Statistics (IBM, Armonk, NY, United States) and *p* < 0.05 was applied as the statistical significance criterion (Cohen, 1992).

## Results

### Pre-test and Post-test

As depicted in Figure 1, there were no significant differences in absolute error between groups in the pre-test (χ^2^ (2) = 3.67, *p* = 0.16, η^2^ = 0.14). All groups improved their scores significantly from pre-test to post-test, amounting to a mean (SD) improvement of 4.77 (5.37) cm in absolute error for 10-year-olds (*Z* = 2.08, *p* = 0.038), 9.18 (5.79) cm for 18-year-olds (*Z* = 2.38, *p* = 0.017), and 7.25 (3.77) cm for adults (*Z* = 2.80, *p* = 0.005). The absolute change in performance from pre-test to post-test, however, was not significantly different amongst the three groups (χ^2^ (2) = 2.96, *p* = 0.23, η^2^ = 0.02). Clearly visible in Figure 1, post-test values were significantly different amongst the groups (χ^2^ (2) = 12.71, *p* = 0.002, η^2^ = 0.49) and post-hoc analysis indicated that the skill levels of both the adults and the 18-year-olds were significantly better than the skill level of 10-year-olds at the post-test (*Z* ≥ 2.69, *p* ≤ 0.007).

### Learning Curves

**FIGURE 2:**
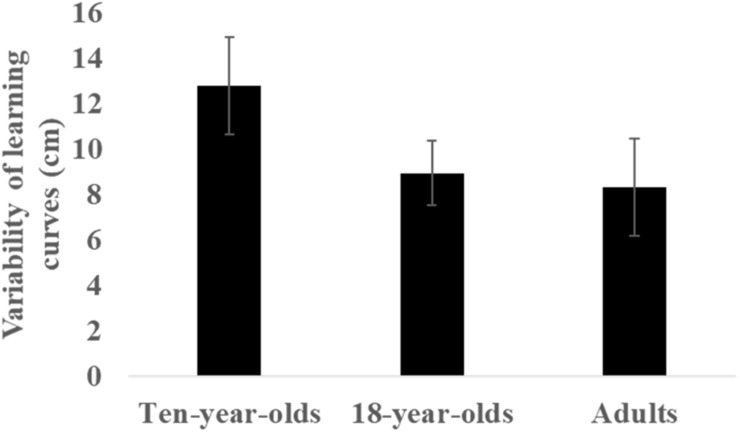
Variability of learning curves (SD) across groups. Bars depict mean and error bars SD.

**FIGURE 3:**
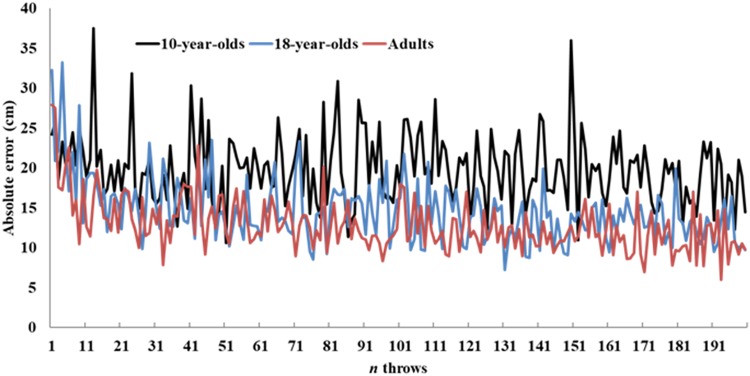
Average learning curves across groups.

As illustrated in Figures 2, 3, there was considerable variability in the learning curves across groups. The 10-year-olds, however, seemed to have higher variability in performance across their 200 throws. Indeed, a Kruskal–Wallis ANOVA indicated significant differences in variability (SD) of learning curves amongst the groups (χ^2^ (2) = 13.45, *p* = 0.001, η^2^ = 0.52). Between-group analysis demonstrated significantly higher learning curve variability in 10-year-olds compared to adults and 18-year-olds (*Z* ≥ 3.08, *p* ≤ 0.002).

**FIGURE 4:**
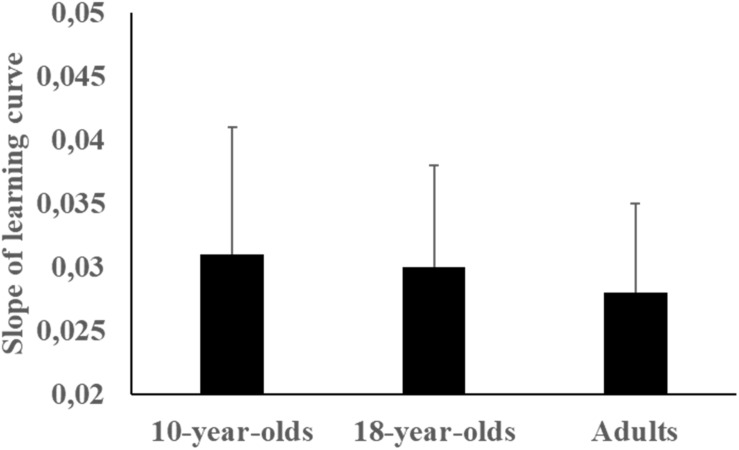
Mean (SD) of slope (regression) of learning curves across study groups.

As can be observed in Figure 3, the average learning curves indicate that the 10-year-olds’ dart-throwing performance decreased at the beginning of the second day, but then improved again towards the end of the day. Nevertheless, the 10-year-olds did show improvement over the period. The learning curves of the 18-year-olds and the adults levelled out somewhat during the transition from the end of day one and the start of day two, before they continued to rise again on day two. Despite the learning curves’ moderate rise – or even no rise whatsoever – none of the test groups ever regressed to baseline levels. Cross-correlation analysis indicated that the 10-year-olds’ average learning curve was significantly similar to the 18-year-olds’ (*r* = 0.54, *p* < 0.01) and the adults’ (*r* = 0.53, *p* < 0.01) average learning curves. Higher and significant similarity was found between the adults’ average learning curve and the 18-year-olds’ average learning curve (*r* = 0.86, *p* < 0.01). However, Kruskal–Wallis ANOVA indicated no significant differences in slope (regression) of learning curves between groups (χ^2^ (2) = 2.37, *p* = 0.32, η^2^ = 0.09, see Figure 4) as well as no significant differences in absolute error at midday I (trials 50–60), end-of-day I (trials 90–100), beginning-of-day II (trials 100–110), and midday II (trials 150–160) (χ^2^ (2) ≤ 3.23, *p* > 0.05, η^2^ = 0., see Figure 1).

## Discussion

The present results do not support the idea of a *golden age* for motor skill learning, as there was no significant difference between any of the three groups with respect to an absolute increase in skill level from the pre-test to the post-test. All three groups improved in skill (Figure 1) and to a similar degree. In addition, the 10-year-olds’ learning curve was more variable compared with those of the remaining groups. While these results should be interpreted with some caution, because of the relatively small groups, they would seem to add support to the argument that 10-year-old children are not residing within a *golden age* for motor skill learning. In contrast to Lee et al.’s (2018) findings, our children showed larger variability compared with the adults. However, this could be interpreted more as a lack of a movement repertoire, demonstrating unintentional variability.

Based on informal observations during the practice sessions, a few interesting points could be raised. The 10-year-olds seemed to lose concentration slightly earlier than the other two test groups. This may in part explain their slightly poorer performance at the post-test compared with those of their older counterparts, as focus and motivation might affect an individual’s ability to learn (Wulf et al., 2010). It is possible that the lapse in concentration could be due to lack of feedback, and one could imagine that it would have been easier for the 10-year-olds to keep up their concentration had they received feedback. However, as noted, earlier, all three groups performed similarly throughout the practice period, and did not perform differently from each other at any point until the post-test at the end of day two (see Figure 1).

Furthermore, it seemed that some of the 10-year-olds were not able to learn from experience which technique would be the most effective. When an adult or an 18-year-old found a throwing strategy that worked, they continued to use this technique for the remainder of the test period. When 10-year-olds found a good strategy, they would typically continue using this until they had missed the target by some margin once, after which they would again alter their strategy. Thus, their throwing continued to be very variable. This may indicate that the 10-year-olds do not have the same ability to understand what has just been successful, or it may simply be due to poor concentration.

In many previous studies of motor-sequence learning (Meulemans et al., 1998; Thomas and Nelson, 2001; Schmitz and Assaiante, 2002; Thomas et al., 2004; Savion-Lemieux et al., 2009), the reported differences between groups may seem to have been caused by the fact that adults performed near floor-level already at the pre-test, meaning that there was little room for improvement. The children, however, started out from skill levels that gave ample room for improvement and, consequently, improved more. Previous experience with the task, or with similar tasks, may have caused adults to reach the floor level in the aforementioned studies. This was already indicated by the fact that they performed much better than the children did at baseline. In addition, experience may have played a role in Emanuel et al. (2008) as participants threw darts with their dominant hand, since the adults probably had much more experience with this particular task. Furthermore, experience, together with cognitive superiority, may have given the adults the advantage of understanding the task in Schärli et al.’s (2013) slackline balancing study, thus being able to figure out an effective strategy of keeping the body steady. In the present study, everyone had similar (non-) experience with performing the exact (left-handed) task.

The present study, considering its rather limited time-span and relatively few participants, lacked the scope of testing the wholesale concept of a *golden age* of motor skill learning. Rather, we wanted to point to how participants’ baseline skill levels will affect how readily they learn skills (as also inspired by Lukács and Kemény, 2015). Furthermore, we wished to highlight that when comparing individuals of different baseline skills, the results may show differences in favor of the group that starts the lowest on a learning curve (usually the children). Thus, it may appear that they have an advantage. In fact, we would argue, based not only on the present results but also on the lack of support for the concept from the remainder of the literature after re-examining the results in the new light, that the *golden age* may be an artefact of inadequately controlled variables in published studies, spurred on by anecdotal evidence. Such anecdotal evidence from sports about the rapid learning of motor skills in relatively young children may well reflect the fact that children spend much more time practicing motor skills (often referred to as technical skills) during their early years within the sport (Van Rossum, 2000), hence they would learn more and faster. Furthermore, the mentioned effect of starting low on a learning curve should not be underestimated.

Our aim was not to prove that learning is not different across the different age groups as this would not be possible within the chosen parameters. We aimed rather at demonstrating that when performing similarly at baseline, and performing, relatively the same task, the learning might not be so dissimilar. Thus, baseline skill levels should be accounted for when comparing motor learning in children and adults – or in any two groups for that matter.

The present study included more repetitions than had many previous ones, perhaps most notably Emanuel et al. (2008), who also used dart throwing as the task. Still none of the present participants came close to demonstrating a floor effect, which the adults had done in several previous studies spanning several tasks (Meulemans et al., 1998; Thomas and Nelson, 2001; Schmitz and Assaiante, 2002; Thomas et al., 2004; Savion-Lemieux et al., 2009). The present study also made a greater effort to ensure that all participants completed the exact same number of repetitions during the test period. Furthermore, the skill levels at baseline were better aligned than in previous studies (Meulemans et al., 1998; Thomas and Nelson, 2001; Schmitz and Assaiante, 2002; Thomas et al., 2004; Watanabe et al., 2007; Savion-Lemieux et al., 2009; Schärli et al., 2013). The alignment of the skill levels in this study was due to the task being scaled so that it was equally novel and similarly challenging for all participants. Although the present results cannot, of course, discard the motor *golden age*, they provide no support for the existence of a motor *golden age* that includes 10-year-olds. Furthermore, they highlight the importance of starting out from similar baselines when comparing motor learning across different age groups.

## Conclusion

The present results showed no significant differences between the change in either absolute error or the learning curves amongst the three age groups. In addition, the learning curves of the children were more variable than those of both older groups. Although the present results should be interpreted with some caution due to the relatively small group sizes, they indicate that when individuals of different ages practice relatively the same skill and start from the same baseline skill level, their learning may not be so dissimilar. Together with the reviewed results from previous studies comparing motor skill learning across different age groups, the present results lend no support to the argument that there is a *golden age* for motor skill learning which includes 10-year-olds.

## Data Availability Statement

The datasets generated for this study are available on request to the corresponding author.

## Ethics Statement

The studies involving human participants were reviewed and approved by the Norwegian Social Science Data. Written informed consent to participate in this study was provided by the participants and the participants’ legal guardian/next of kin where appropriate.

## Author Contributions

MS, HL, and AP conceived the idea and designed the experiment. MS collected the data and analyzed it together with HL. MS wrote the first draft of the manuscript. All authors made substantial contributions to the final manuscript and approved it for publication.

## Conflict of Interest

The authors declare that the research was conducted in the absence of any commercial or financial relationships that could be construed as a potential conflict of interest.
